# The winking sign is an indicator for increased femorotibial rotation in patients with recurrent patellar instability

**DOI:** 10.1007/s00167-022-06971-y

**Published:** 2022-04-19

**Authors:** Andreas Flury, Sandro Hodel, Julian Hasler, Esfandiari Hooman, Sandro F. Fucentese, Lazaros Vlachopoulos

**Affiliations:** 1 Department of Orthopedics, Balgrist University Hospital, University of Zurich, Forchstrasse 340, 8008 Zurich, Switzerland; 2 Research in Orthopedic Computer Science (ROCS), Balgrist University Hospital, University of Zurich, Zurich, Switzerland

**Keywords:** Femorotibial rotation, Knee rotation, Patellar instability, Winking sign

## Abstract

**Purpose:**

Rotation of the tibia relative to the femur was recently identified as a contributing risk factor for patellar instability, and correlated with its severity. The hypothesis was that in patellofemoral dysplastic knees, an increase in femorotibial rotation can be reliably detected on anteroposterior (AP) radiographs by an overlap of the lateral femoral condyle over the lateral tibial eminence.

**Methods:**

Sixty patients (77 knees) received low-dose computed tomography (CT) of the lower extremity for assessment of torsional malalignment due to recurrent patellofemoral instability. Three-dimensional (3D) surface models were created to assess femorotibial rotation and its relationship to other morphologic risk factors of patellofemoral instability. On weight-bearing AP knee radiographs, a femoral condyle/lateral tibial eminence superimposition was defined as a positive winking sign. Using digitally reconstructed radiographs of the 3D models, susceptibility of the winking sign to vertical/horizontal AP knee radiograph malrotation was investigated.

**Results:**

A positive winking sign was present in 30/77 knees (39.0%) and indicated a 6.3 ± 1.4° increase in femorotibial rotation (*p* < 0.001). Femoral condyle/tibial eminence superimposition of 1.9 mm detected an increased femorotibial rotation (> 15°) with 43% sensitivity and 90% specificity (AUC = 0.72; *p* = 0.002). A positive winking sign (with 2 mm overlap) disappeared in case of a 10° horizontally or 15° vertically malrotated radiograph, whereas a 4 mm overlap did not disappear at all, regardless of the quality of the radiograph. In absence of a winking sign, on the other hand, no superimposition resulted within 20° of vertical/horizontal image malrotation. Femorotibial rotation was positively correlated to TT–TG (*R*_2_ = 0.40, *p* = 0.001) and patellar tilt (*R*_2_ = 0.30, *p* = 0.001).

**Conclusions:**

The winking sign reliably indicates an increased femorotibial rotation on a weight-bearing AP knee radiograph and could prove useful for day-by-day clinical work. Future research needs to investigate whether femorotibial rotation is not only a prognostic factor but a potential surgical target in patients with patellofemoral disorders.

**Level of evidence:**

III.

## Introduction

Patellofemoral disorders can be caused by a variety of bony deformities of the lower limb, acting either alone or in combination. In fact, given the positive relationship between femoral and tibial torsion, trochlear dysplasia, frontal mechanical axis, and tibial tuberosity–trochlear groove (TT–TG) distance [20, 27], abnormal bony geometry is only rarely limited to one parameter. Current evidence shows that especially increased femoral antetorsion (FT) and valgus alignment promote lateral patellar instability. Thus, isolated reconstruction of the medial patellofemoral ligament (MPFL) was suggested to be insufficient in case of higher degrees of FT [25]. Because excessive FT negatively affects the outcome after surgical treatment for patellofemoral instability [13], recent studies performed osteotomies to correct axial and frontal plane malalignment with good results [6, 10, 12, 14, 19]. However, the validity of FT as the sole indicator for functional performance is questioned [33, 35].

Recently, a previously rather unknown morphologic feature that might play a role in the pathophysiology of patellar instability has received attention: The relative rotation of the femur on the tibia [2, 28]. Assessed on MRI, the highest values of femorotibial rotation through the knee joint were detected in patients with a fixed/chronic patellar dislocation, followed by standard traumatic instability, and controls [28]. To date, the dynamic impact or functional relevance of increased femorotibial rotation remains unclear. Further insight into femorotibial rotation is needed, as it may become not only a prognostic factor but a potential surgical target in patients with patellofemoral disorders.

The purpose of the present study was to raise awareness of torsional knee malalignment by simplifying the diagnosis of femorotibial rotation. Therefore, it was hypothesized that an increase in femorotibial rotation can be reliably detected not only on MRI or CT scans but also on conventional weight-bearing radiographs of the knee by an overlap of the lateral femoral condyle over the lateral tibial eminence. Three-dimensional (3D) surface models were generated to test the diagnostic performance of this radiographic phenomenon, and further investigate the relationship of femorotibial rotation to other morphological factors such as TT–TG and patellar tilt. The goal of this paper is to introduce the winking sign as a screening tool for day-to-day clinical work to encourage and facilitate future research in this field of expertise.

## Materials and methods

This study was approved by the Institutional Review Board and the ethical committee (Zurich Cantonal Ethics Commission, KEK 2021-01428). It was conducted entirely at the authors' institution.

### Study cohort

**Fig. 1:**
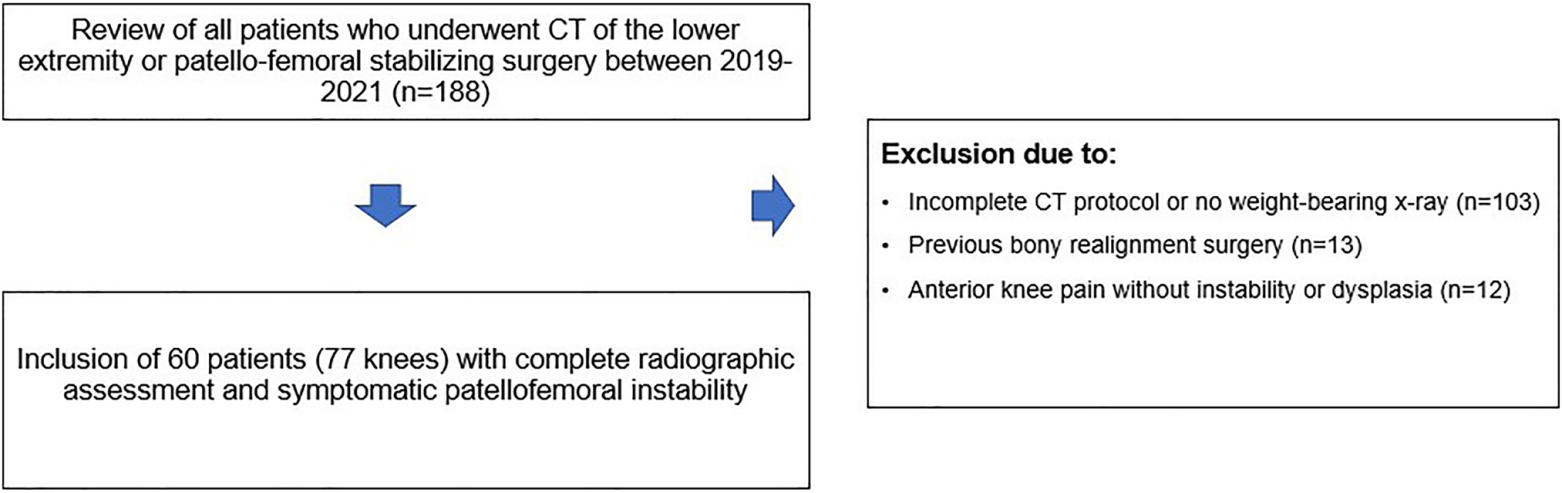
Flowchart and eligibility. CT: Computed tomography

A retrospective review was conducted on 188 patients who received low-dose computed tomography (CT) scans of the lower extremity for the assessment of torsional malalignment at the authors’ institution from 2019 to 2021. All CTs were screened for signs of patellofemoral dysplasia [8], and classified according to Dejour et al. [4]. Patients with incomplete radiographic workup (e.g., missing weight-bearing long leg radiograph) (*n* = 103), previous bony realignment surgery (*n* = 13), or anterior knee pain without patellofemoral instability or with end-stage patellofemoral osteoarthritis [32] (*n* = 12) were excluded. Finally, 60 patients (77 knees) were eligible for analysis with a median age of 21.8 ± 6.1 years (range 12–40 years) including 34 female patients (56.7%) (Fig. 1).

### Creation and analysis of three-dimensional surface models

**Fig. 2:**
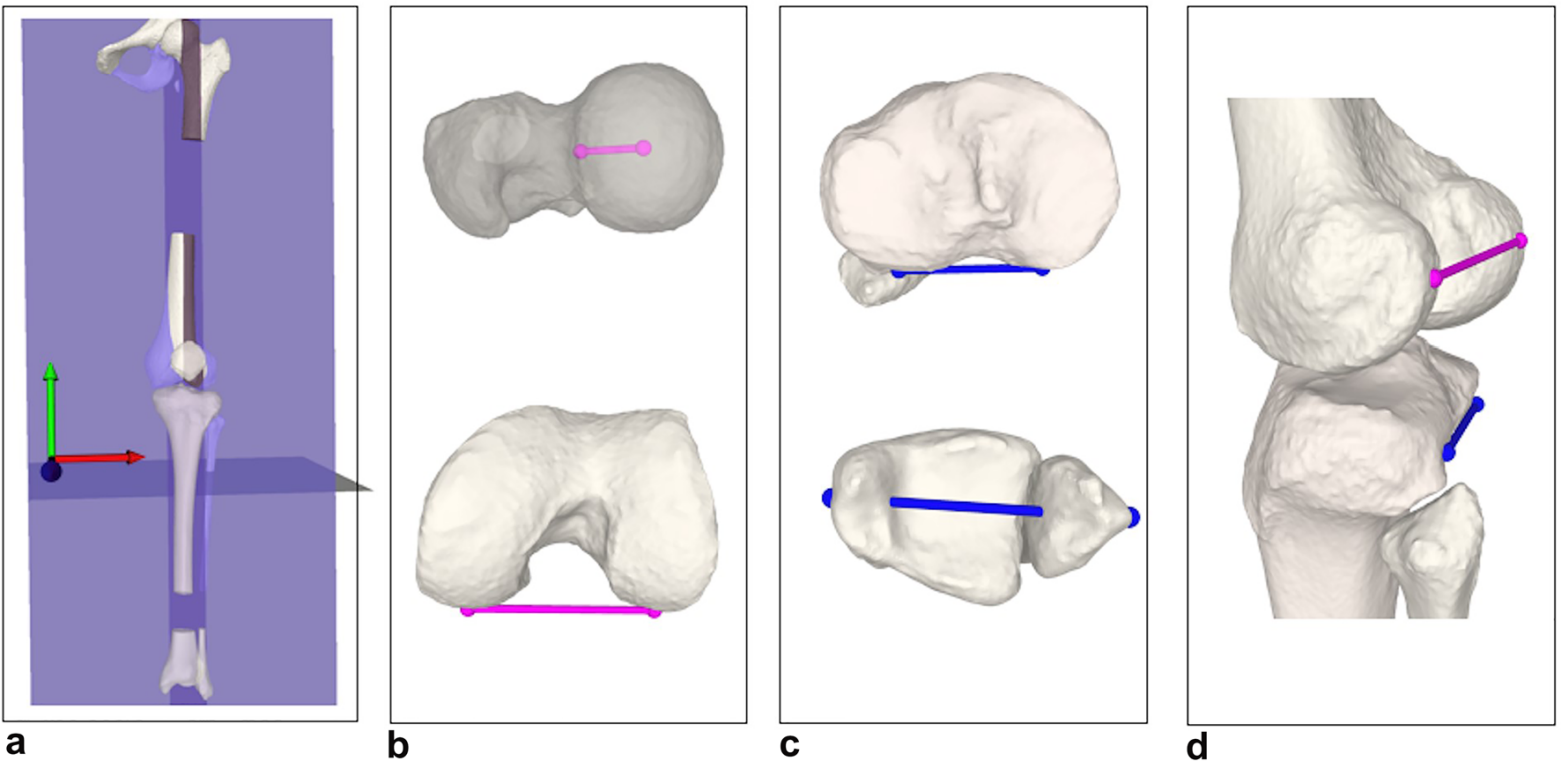
Three-dimensional axial leg alignment and femorotibial rotation measurement. **A** Orientation of coordinate system. Femoral torsion, tibial torsion, and femorotibial rotation were all measured as 2D angles projected onto the axial plane (plane normal = $\overrightarrow{\mathrm{y}}$, green). **B** Femoral antetorsion was measured between femoral neck axis (top) and posterior femoral condyle axis (bottom), both pink. **C** Tibial torsion was measured between proximal tibia axis (top) and malleolar axis (bottom), both blue. **D** Femorotibial rotation was measured between posterior femoral condyle axis (pink) and proximal tibia axis (blue)

All patients underwent preoperative supine CT scan of the lower extremity according to a standardized protocol including all anatomical structures of interest: (hip center, proximal femur, knee center with distal femur and proximal tibia, and ankle joint center with distal tibia, distal fibula, and talus). The hip center was defined as the center of a sphere, fitted to the femoral head. The knee center was defined as the midpoint between the intercondylar eminences on the tibial plateau. The ankle center was determined as the center of the distal articular surface of the tibia and fibula [34]. 3D surface models of the lower extremities were created using global thresholding segmentation and region growing using the MIMICS software (MIMICS, Materialize, Belgium) and imported into an in-house developed surgical planning software CASPA (Balgrist Zurich, Switzerland). A 3D coordinate system was defined according to International Society of Biomechanics (ISB) [38]. Hip–knee–ankle angle (HKA) [15, 34], femoral [17, 23] and tibial torsion [16, 18, 22], and TT–TG [18, 22] were measured on each 3D model according to the previously described and validated methods (summarized in Fig. 2). Femorotibial rotation was calculated as the projected 2D angle in the axial plane between the distal femoral axis and the proximal tibial axis both defined for the previously described femoral and tibial torsion measurements (positive values indicating a relative tibial external rotation) (Fig. 2). Patellar tilt was measured in 2D axial CT slices according to Detour et al. [5] with reference to the posterior condyles given that a standardize 3D measurement is missing in the available literature.

Patellar tilt was measured by two observers blinded to the outcome in a picture archive and communication system (Phönix PACS v. 5.8.1, Germany). Reliability testing for the 3D measurements was not repeated due to the utilization of a semi-automatic measurement procedure and given that excellent reliability was reported in several of the aforementioned original publications.

### Quantification of femorotibial rotation in weight-bearing radiographs

**Fig. 3:**
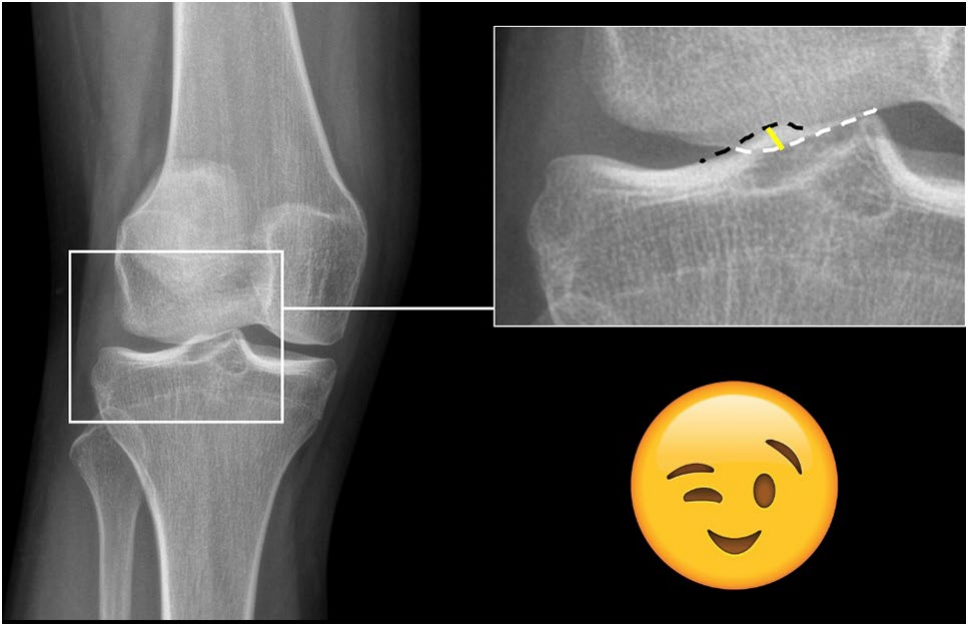
Definition of the winking sign and measurement of the overlap of the lateral femoral condyle and the lateral tibial eminence. Left: Example of a positive winking sign in a female patient with femorotibial torsion of 22°. Right (magnification): An overlap of the femoral condyle (white dotted line) with the lateral tibial eminence (black dotted line) defined a positive winking sign. The femoral condyle overlap was measured perpendicular to the lateral tibial eminence at the location of the greatest overlap (yellow line) in mm

To quantify the magnitude of femorotibial rotation in weight-bearing AP knee radiographs, the overlap of the lateral condyle and the lateral tibial eminence was measured in millimeters perpendicular to the cortex of the lateral tibial eminence (Fig. 3). The presence of an overlap of the femoral condyle was defined as a positive winking sign, as the obliterated lateral joint space appears similar to a winking eye.

### Robustness of the winking sign

The robustness of the winking sign with respect to rotation and tilt of the radiograph (vertical and horizontal malrotation) was assessed with digitally reconstructed radiographs (DRR) [9]. This was performed on one patient with a negative winking sign, a second patient with a 2 mm condylar overlap, and a third patient with a 4 mm condylar overlap. Starting from a true anteroposterior (AP), 3D models were tilted in both directions as well as internally/externally rotated in 5° steps up to 10° and synthetic radiographs (i.e., DRRs) were generated from those angles respectively.

Similar to the patellar tilt, the presence of a winking sign (yes/no) as well as the absolute value of femoral condyle/tibial eminence superimposition (in mm) was measured by two observers blinded to the outcome for calculation of inter- and intra-reader reliability. True AP knee images were defined as follows: femoral/tibial condyles symmetrical, fibular head slightly superimposed by the lateral tibial condyle, and the patella centered. Measurement accuracy per pixel was 0.1 mm and 0.1°. Regarding clinical relevance, outcome variables are given in one decimal.

### Statistical analysis

An a priori power analysis (*α* = 0.05, power level *β* = 0.80) revealed a minimum sample size of *n* = 44 (22 per group) to detect a minimum increase of 5° in femorotibial rotation with a positive winking sign, assuming a mean femorotibial rotation of 8.2 ± 6.5° according to[1]. The power analysis was conducted using G*Power (version 3.1; Franz Faull, Universität Kiel).

Normal distribution of the data was tested with the Shapiro–Wilk test. The herein data are reported as mean ± standard deviation (SD) or as counts (percentages). The inter-reader reliability of the winking sign was assessed using Cohen’s kappa (*κ*). The inter-reader and intra-reader reliability for patellar tilt and amount of femoral condyle overlap was assessed using intraclass-correlation coefficients (ICC) and a two-way mixed-effect model assuming a single measurement and absolute agreement.

Continuous variables between patients with and without a winking sign and between genders (due to gender-related differences in laxity) were analyzed with an unpaired Student’s t test or Mann–Whitney U test, as appropriate. Differences between categoric values were analyzed using Pearson's Chi-square test.

The diagnostic performance of the lateral condyle overlap (in mm) was analyzed using a receiver-operating characteristic (ROC) curve to detect a femorotibial rotation > 15° (according to [1]: average knee rotation angle plus one standard deviation on CT/MRI in a patellofemoral dysplastic cohort). Area under the curve (AUC), sensitivity, specificity, and cut-off were reported. The influence of femorotibial rotation (in °) on TT–TG distance and patellar tilt was analyzed in a linear regression model and reported as *R*-squared (*R*^2^).

To identify potential confounders that influence femorotibial rotation, correlations between HKA, FT, tibial torsion, age, sex, BMI, and femorotibial rotation (°) were analyzed using Spearman's rank test. The significance was set < 0.05. Data were analyzed with SPSS version 26 (SPSS Inc, Chicago, IL, USA).

## Results

Identifying a positive winking sign demonstrated perfect agreement (*κ* = 1.00) (*p* < 0.001). The inter-reader reliability of femoral condyle/tibial eminence superimposition and patella tilt demonstrated an ICC of 1.00 (95% CI 0.99–1.00) (*p* < 0.001) and 1.00 (95% CI 1.00–1.00) (*p* < 0.001), respectively. The intra-reader reliability was 1.00 (95% CI 1.00–1.00) (*p* < 0.001) for both.

**Fig. 4:**
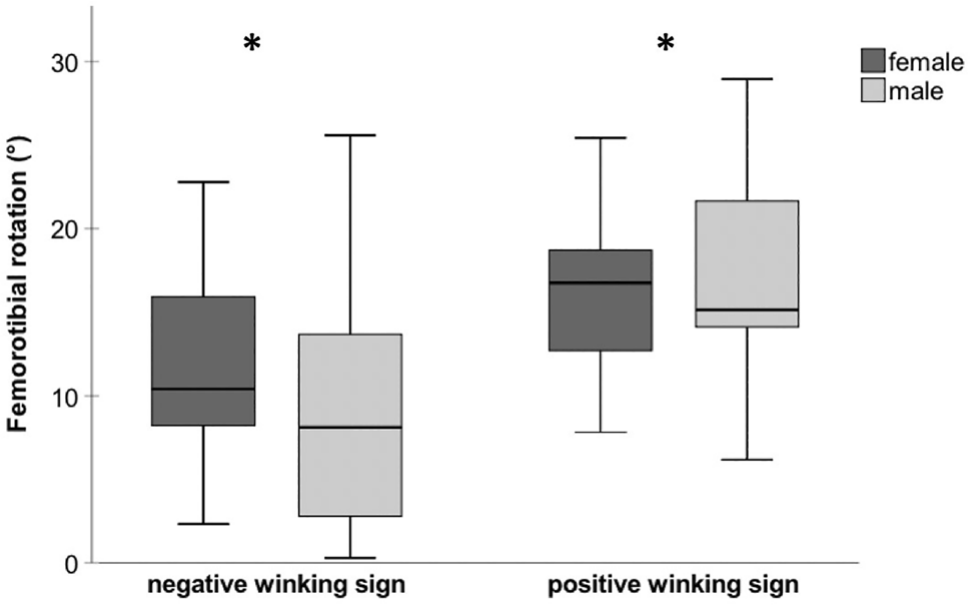
Femorotibial rotation according to the presence of a winking sign and gender. Boxplots depicts median (line), IQR (box), and minimum and maximum (whisker) of femorotibial rotation (°) according to the presence of the winking sign and gender. Asterisks depict a significant increase of femorotibial rotation in patients with a positive winking sign (*p* < 0.001) without significant gender differences (*p* = 0.346)

A positive winking sign was present in 30 knees (39.0%) and indicated an increase of 6.3 ± 1.4° in femorotibial rotation compared to patients without any overlap (p < 0.001) (Table 1 and Fig. 4).

**Table 1:** Differences among patients with and without a winking sign

	Negative winking sign 47 knees (100%)	Positive winking sign 30 knees (100%)	*p* value
Age (years)	21.3 ± 6.0	22.4 ± 6.4	n.s.*
BMI (kg/m^2^)	26.4 ± 5.7	25.3 6.3	n.s
Female gender *n* (%)	24 (51.1)	17 (56.7)	n.s
Trochlea dysplasia: n (%)			**0.035**
Dejour type A	5 (10.6)	0 (0)	
Dejour type B	15 (31.9)	6 (20.0)	
Dejour type C	13 (27.7)	6 (20.0)	
Dejour type D	14 (29.8)	18 (60.0)	
HKA (°)	-1.4 ± 4.4	0.1 ± 2.3	0.020*
Femoral torsion (°)	18.4 ± 11.6	23.3 ± 8.8	0.051
Tibial torsion (°)	27.3 ± 8.8	32.4 ± 9.6	**0.018**
Femorotibial rotation (°)	10.2 ± 6.1	16.5 ± 5.5	** < 0.001**
TT–TG (mm)	15.9 ± 5.2	19.9 ± 3.4	** < 0.001**
Patellar tilt (°)	28.2 ± 8.6	37.0 ± 11.6	** < 0.001**

Numeric values: *Mann–Whitney *U* test, remaining Student's *t* test. Categoric values: Pearson’s Chi-square test. Significant values marked bold (*p* < 0.05)

**Fig. 5:**
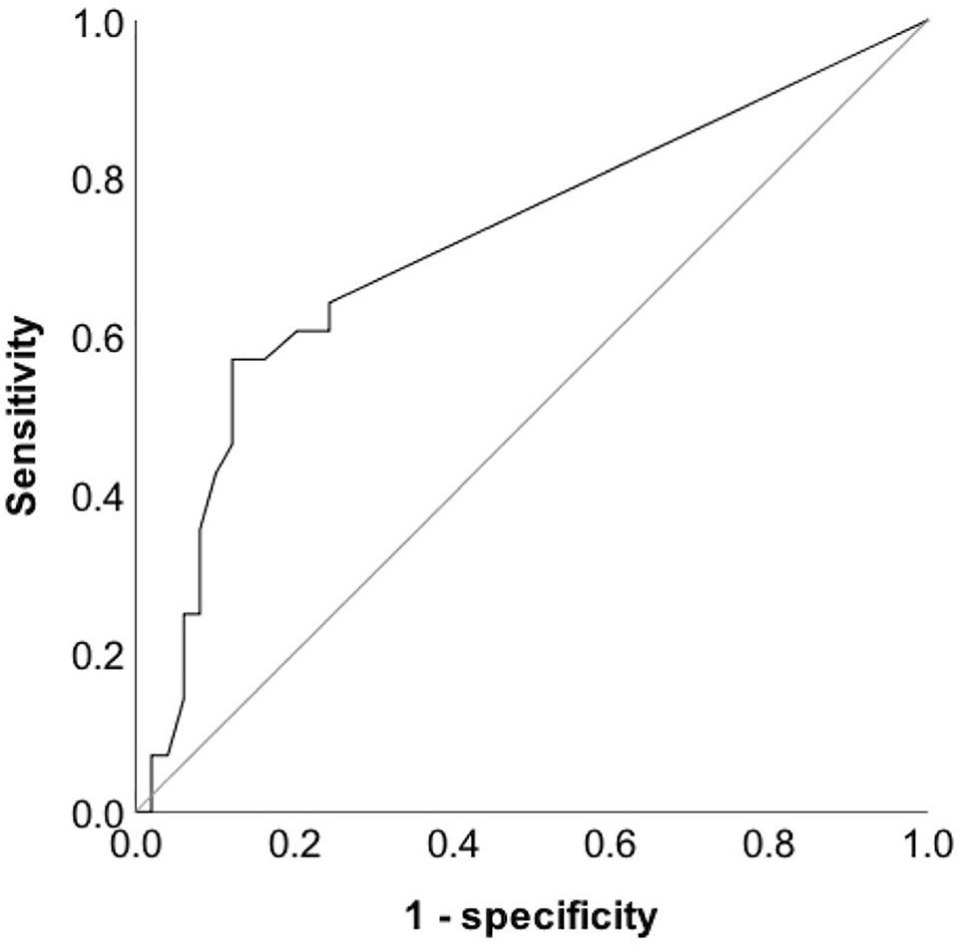
Receiver-operating characteristic curve for the winking sign. Area under the curve = 0.72 (95% CI 0.59–0.84) (*p* = 0.002). Reference line (gray): AUC = 0.5. A cut-off of 1.9 mm lateral femoral condyle overlap detected an increased femorotibial rotation (> 15°) with a sensitivity of 43% and a specificity of 90% (*p* = 0.002)

A 1.9 mm cut-off value for femoral condyle/lateral tibial eminence superimposition detected an increased femorotibial rotation (> 15°) with a 43% sensitivity and a 90% specificity (AUC = 0.72; *p* = 0.002) (Fig. 5).

In case of a positive winking sign, the robustness with respect to malrotation and tilt of the AP knee radiograph revealed the following mean absolute errors of the overlap: 0.9 ± 0.5 mm (range 0.3–1.7 mm) per 5° rotation (increasing overlap with internal rotation, decreasing with external rotation) and 1.5 ± 0.4 (range 1.0–2.8 mm) per 5° of tilting (increasing overlap with upward tilt, decreasing with downward tilt). In the absence of a winking sign in the true AP radiograph, no lateral condyle overlap appeared within the range of 20° malrotation or tilt. A positive winking sign disappeared in 10° upward tilt in the patient with 2 mm overlap, representing a false-negative rate of 6.3%. A pronounced overlap of 4 mm did not disappear at all, regardless of the quality of the radiograph.

**Fig. 6:**
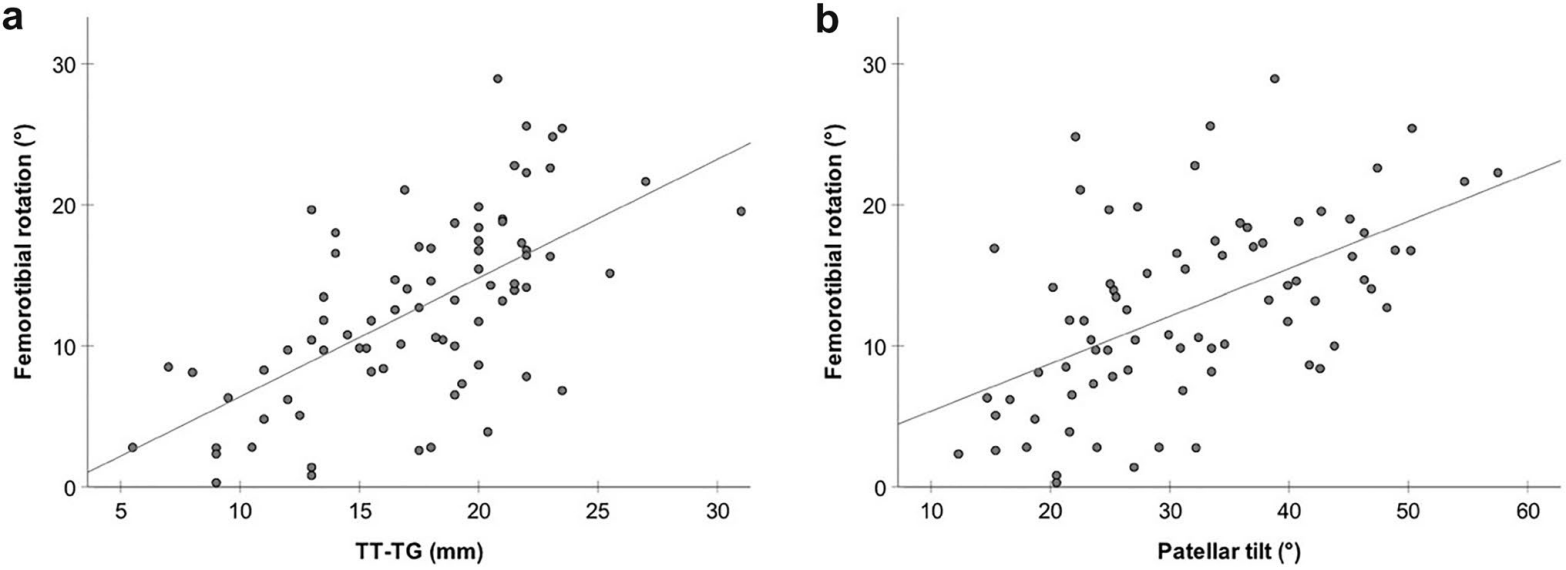
Influence of femorotibial rotation on tibial tuberosity–trochlear groove (TT–TG) distance and patellar tilt. **A** Scatterplot depicts nearly linear relationship between femorotibial rotation and TT–TG (mm), *R*^2^ = 0.40 *p* < 0.001. **B** Scatterplot depicts nearly linear relationship between femorotibial rotation and patellar tilt (°), *R*^2^ = 0.30, *p* < 0.001

An increase in femorotibial rotation led to a nearly linear increase in TT–TG (*R*^2^ = 0.40 p < 0.001) and patellar tilt (*R*^2^ = 0.30 *p* < 0.001) (Fig. 6). No significant influence of age, gender, BMI, and frontal and axial leg alignment on femorotibial rotation was found (*Spearman's rank correlation*).

## Discussion

The most important finding of this study is that one can reliably predict femorotibial rotation based on the presence of a winking sign in weight-bearing AP knee radiographs. However, the winking does not stem from a malrotated AP knee radiograph. Moreover, femorotibial rotation was associated with increased TT–TG and patellar tilt.

A variety of factors alters the mechanics of the patellofemoral joint and increase joint stress, such as genu valgum [11, 30, 31], increased TT–TG distance, trochlear morphology [21, 31], and femoral rotational deformity [7, 11, 24, 26, 29]. The proposition that the rotational profile through the level of the knee joint is an integral part of patellofemoral kinematics is mainly based on the finding of studies conducted on total knee arthroplasty. In relation to patellofemoral stability, the current literature on femorotibial rotation is scarce due to its novelty. Even though it was around for a couple of years, it was only recently named as well as highlighted as the main contributor to the TT–TG value. Next to medialization of the trochlea and lateralization of the tibial tubercle, the TT–TG distance was more strongly affected by the rotation between the tibia and the femur [36, 37]. Only a few studies since then have focused on knee rotation, and reported an increased femorotibial rotation in patients with patellar instability [1, 2]. Relative rotation of the femur on the tibia as a pathoanatomic factor of patellofemoral instability was confirmed in the recent study of Lin et al. [28], where femorotibial rotation on MRI correlated with the severity of patellar instability. In detail, rotational deformity was the highest in patients with a chronically dislocated patella, followed by standard traumatic instability patients, and controls [28].

The purpose of this study was to introduce a simple radiographic sign to detect increased native knee rotation on standard AP radiographs. To check whether the winking sign is not simply a projection phenomenon caused by vertical and horizontal image malrotation, 3D surface models of all knees were created and the robustness was assessed using DRRs. Absolute femoral condyle/lateral tibial eminence overlap was affected by malrotation and tilt of the radiograph. However, the mean absolute overlap error in case of 10° vertical or horizontal image malrotation was small. Furthermore, a winking sign with a 4 mm overlap did not disappear whatsoever, regardless of the quality of the AP knee radiograph. Moreover, in absence of a winking sign in the true knee AP radiograph, no lateral condyle overlap appeared within 20° of image malrotation. Therefore, the presence of a winking sign reliably indicates increased femorotibial rotation. However, a winking sign that is actually positive can be missed in case of a > 10° vertically or horizontally malrotated AP radiograph.

To date, the clinical relevance of femorotibial rotation is unclear. Nevertheless, the relationship to other commonly used measurements in patellar instability such as TT–TG and patellar tilt is evident. In fact, femorotibial malrotation might represent the main pathology in those cases, since not only TT–TG distance but patellar tilt is strongly affected by knee rotation [3, 37]. In general, surgical strategy should be based on individual deformity analysis. In case of increased native knee rotation (but normal femoral and tibial torsion), the posterolateral knee corner could be hypothesized to be a potential surgical target instead of a derotational osteotomy or transfer of the tibial tubercle. If the posterolateral corner benefits from soft-tissue augmentation, so that it exerts enough force to correct tibial rotation in relation to the femur (and concomitantly femoral subluxation on the lateral tibial plateau; see Fig. 2D) will need further investigation.

There is no consensus on what is the normal and what is increased femorotibial rotation. The current study cohort only consisted of patients with recurrent patellofemoral instability. However, Lin et al. [28] found that any external knee rotation was associated with patellar instability. In contrast to 1.6° and 8.5° external femorotibial rotation in patients with traumatic and chronic patellar instability, respectively. Controls averaged 3.8° internal femorotibial rotation. Future studies should investigate the reference point beyond which femorotibial rotation should be considered pathologic and trigger intervention. The main limitation is that diagnostic performance of the winking sign was investigated using weight-bearing radiographs but unloaded CT data. Muscular forces and joint movement (screw-home mechanism) under weight-bearing conditions might bias the correlation due to a potential dynamic factor of knee rotation. In case of a potential soft-tissue pathology, knee rotation could be influenceable through muscle contraction, foot stance, or hip rotation. Moreover, we have not investigated the impact of knee flexion on femoral condyle/lateral tibial eminence superimposition. Native knee rotation was measured on CT and, thus, in extension. According to the literature, the position of the tibia relative to the femur changes during flexion [39]. However, the previous studies oppose the effect of knee flexion on knee rotation [1], possibly due to an altered compensatory (screw-home) mechanism in patients with patellofemoral instability[36]. Therefore, if the weight-bearing knee X-ray is performed properly, no false-positive overlap should be expected. Overall, the winking sign seems to be sufficient for screening purposes, and therefore useful in the day-by-day clinical work. Its presence indicates a mean of 6.3° femorotibial rotation, and in case of an overlap of > 2 mm a femorotibial rotation of > 15°. Nevertheless, further research is needed to answer questions concerning the dynamic nature of the pathology.

## Conclusion

The winking sign is not the result of vertical and horizontal AP knee radiograph malrotation and therefore reliably indicates increased femorotibial rotation in patients with patellar instability. Therefore, the winking sign represents a useful screening tool in the day-by-day clinical work. However, future research needs to evaluate whether femorotibial rotation is not only a prognostic factor but a potential surgical target in patients with patellofemoral disorders.

## Data Availability

The datasets used and analyzed during the current study are available from the corresponding author on reasonable request.
